# Candidate Genes as Biomarkers in Lipopolysaccharide-Induced Acute Respiratory Distress Syndrome Based on mRNA Expression Profile by Next-Generation RNA-Seq Analysis

**DOI:** 10.1155/2018/4384797

**Published:** 2018-04-08

**Authors:** Qi-Quan Wan, Di Wu, Qi-Fa Ye

**Affiliations:** ^1^Department of Transplant Surgery, The Third Xiangya Hospital, Central South University, Changsha 410013, China; ^2^Zhongnan Hospital of Wuhan University, Institute of Hepatobiliary Diseases of Wuhan University, Transplant Center of Wuhan University, Hubei Key Laboratory of Medical Technology on Transplantation, Wuhan, Hubei 430071, China

## Abstract

Up until now, the regulation mechanism at the level of gene during lipopolysaccharide- (LPS-) induced acute respiratory distress syndrome (ARDS) remains unclear. The discovery of differentially expressed genes (DEGs) between LPS-induced ARDS rats and normal rats by next-generation RNA sequencing analysis is of particular interest for the current study. These DEGs may help clinical diagnosis of ARDS and facilitate the selection of the optimal treatment strategy. Randomly, 20 rats were equally divided into 2 groups, the control group and the LPS group. Three rats from each group were selected at random for RNA sequencing analysis. Sequence reads were obtained from Illumina HiSeq4000 and mapped onto the rat reference genome RN6 using Hisat2. We identified 5244 DEGs (Fold_Change > 1.5, and *P* < 0.05) in the lung tissues from LPS-treated rats compared with normal rats, including 1413 upregulated and 3831 downregulated expressed genes. Lots of chemokine family members were among the most upregulated genes in LPS group. Gene ontology (GO) analysis revealed that almost all of the most enriched and meaningful biological process terms were mainly involved in the functions like immune-inflammation response and the pathways like cytokine-cytokine receptor interaction. We also found that, as for GO molecular function terms, the enriched terms were mainly related to chemokines and cytokines. DEGs with fold change over 100 were verified by quantitative real-time polymerase chain reaction and reanalyzed by gene-gene coexpression network, and the results elucidated central roles of chemokines in LPS-induced ARDS. Our results revealed some new biomarkers for uncovering mechanisms and processes of ARDS.

## 1. Introduction

The acute respiratory distress syndrome (ARDS) is a life-threatening diffuse lung disease owing to direct or indirect lung injury factors, such as pneumonia, severe sepsis, aspiration, drug toxicity, and multiple blood transfusion [1]. It is characterized by an excessive lung inflammatory response, which can lead to the increased alveolar-capillary permeability, diffused pulmonary interstitial and alveolar edema, and impaired gas exchange functions and reduced alveolar fluid clearance of the lungs with consequent hypoxemia [1–3]. The main pathological processes of ARDS include the destruction of the alveolar-capillary unit, the collapse of alveolar, the flooding of the alveolus with a proteinaceous exudate, the release of inflammatory cytokines and chemokines, and lung neutrophilia [4].

Over the past decade, considerable work has been done for testing the contribution of genetic factors correlated with ARDS, including those genes for B-cell lymphoma 2-associated agonist of cell death, the angiopoietin-2, topoisomerase 2-alpha, cytoplasmic cyclins B1 and B2, peptidase inhibitor 3, olfactomedin 4, lipocalin 2, CD24, bactericidal/permeability-increasing protein, and mannose binding lectin-2 [5–10]. Furthermore, based on the gene expression profiling on peripheral blood samples from ARDS patients, Dolinay and colleagues demonstrated that the inflammasome pathway and its downstream cytokines play pivotal roles in ARDS development [11]. Although some genes were demonstrated to be associated with ARDS, currently little is known regarding the regulation mechanism at the level of gene, leading to the lack of an effective therapeutic method for severe cases [12–15].

The most common causes of ARDS are bacterial pneumonia and sepsis, wherein Gram-negative bacteria are a prominent cause. Lipopolysaccharide (LPS) is the major constituent of the outer envelope of all Gram-negative bacteria. It induces the injury of epithelial cells along with resident alveolar macrophages in the airway, thereby further resulting in a cascade of events including production of cytokines and chemokines, recruitment of neutrophils, monocytes, and lymphocytes into the alveolar space [16, 17]. RNA sequencing (RNA-Seq) analysis facilitates the ability to look at the profiling of the transcriptomes and has become an invaluable tool in different areas of biology [18].

However, mRNA expression profile in lung tissues from rats with LPS-induced ARDS is not clear up until now. Thus, based on mRNA expression profile, the analysis of potential genes and pathways related to LPS-induced ARDS may be a breakthrough for the further understanding of ARDS pathological mechanism, clinical diagnosis, and the choice of optimal treatment strategy.

The present study detected the changes in the mRNA expression profiles of lung tissues from rats with LPS-induced ARDS rats compared with normal rats. To our best knowledge, this is the first study to investigate the mRNA expression profiling in lung tissues from rats with LPS-induced ARDS using means of RNA-Seq.

## 2. Material and Methods

### 2.1. Reagents

LPS (*Escherichia coli* serotype 055:B5) was purchased from Sigma-Aldrich (St Louis, MO, USA).

### 2.2. Animals

Male specific pathogen-free Sprague-Dawley rats with a body weight of 200–250 g from Hunan SJA Laboratory Animal Co., Ltd. (Changsha, China), were kept for 1 wk under controlled temperature and humidity with a regular day-night cycle, with free access to standard laboratory food and water.

### 2.3. Ethics

The rats were authorized by the Inspection of the Institutional Animal Care and Use Committee of the Third Xiangya Hospital of Central South University (IRB number LLSC(LA)2017-012).

### 2.4. Model of ARDS Induced by LPS and Sample Collections

Randomly, 20 rats were equally divided into 2 groups, the control group and the LPS group. ARDS was induced by LPS as our previous report [19]. Rats were sacrificed for lung collection at 7 h after challenge.

### 2.5. Histological Examination

The lung specimens were harvested from rats and immediately fixed in 10% paraformaldehyde for 24 h. Then, they were embedded in paraffin and cut (5 *μ*m sections) for histology. Next, the sections were stained with hematoxylin and eosin before being photographed and analyzed by light microscopy.

### 2.6. RNA Isolation, Transcriptome Library Preparation, and Sequencing

Three fragmented frozen lung tissue samples were randomly selected from each group for the RNA isolation in 3 replicates. Total RNAs were extracted using TRIzol™ Reagent (Invitrogen, Carlsbad, CA, USA) following the manufacturer's instructions and random primers were used to generate complementary DNA. After quantitative analysis and quality inspection, KAPA-Stranded RNA-Seq Library Prep Kit (Illumina) was used to construct sequencing libraries. Sequencing was carried out using an Illumina HiSeq 4000 Sequencing System for 150 cycles. After the data preprocessing, gene level fragments per kilobase of exon per million fragments mapped [20] and significant changes in gene and transcript expression were then calculated using Ballgown software [21].

### 2.7. Bioinformatics Analysis

Enrichment of gene ontology (GO) terms was measured [22, 23]. Further, pathway analysis for the differentially expressed genes (DEGs) was carried out by Kyoto Encyclopedia of Genes and Genomes (KEGG) tool which was performed by STIRNG analysis (https://string-db.org/) [22]. The interactions of the DEGs were also determined by STRING.

### 2.8. qRT-PCR

Validation of genes was performed using quantitative real-time polymerase chain reaction (qRT-PCR) (in triplicate) via ViiA 7 Real-Time PCR System (Applied Biosystems) in all 3 RNA samples from each group. The primers for genes selected to be verified and the housekeeping gene *β*-actin were synthesised by KangChen Bio-Tech (Shanghai, China). Primers sequences are displayed in Table S1. Data were analyzed by the comparative Ct method based on the expression of *β*-actin.

### 2.9. Statistical Analysis

All results were expressed as the mean ± standard deviation. The thresholds for DEGs were fold change (FC) > 1.5 and *P* < 0.05. Data were analyzed using the ArrayStar V4.1 (DNASTAR, Madison, WI) software for generation of the heat maps of DEGs. The data was analyzed by the analysis of variance for the relative quantitative expressions of the genes by qRT-PCR, followed by Student-Newman-Keuls post hoc tests using the SPSS Statistics 19.0 software package (IBM, Chicago, IL, USA).

## 3. Results

### 3.1. Histopathological Evaluation

The effect of LPS on rat lung tissues was evaluated (Figure S1). In the LPS group, lung tissues were significantly damaged, with alveolar edema, thickening of the alveolar wall, and infiltration of inflammatory cells. The appearances above demonstrated the success in establishing the model of ARDS.

### 3.2. Analysis of Composition and Depth of the Transcriptional Profiles Obtained

The study included RNA samples extracted from lung tissue samples of 3 ARDS rats and 3 normal rats. For each RNA sample transcriptome libraries were constructed and sequenced using RNA-Seq. The obtained reads were filtered by quality and mapped onto rat genome version RN6.

### 3.3. Sample Clustering

**Figure 1:**
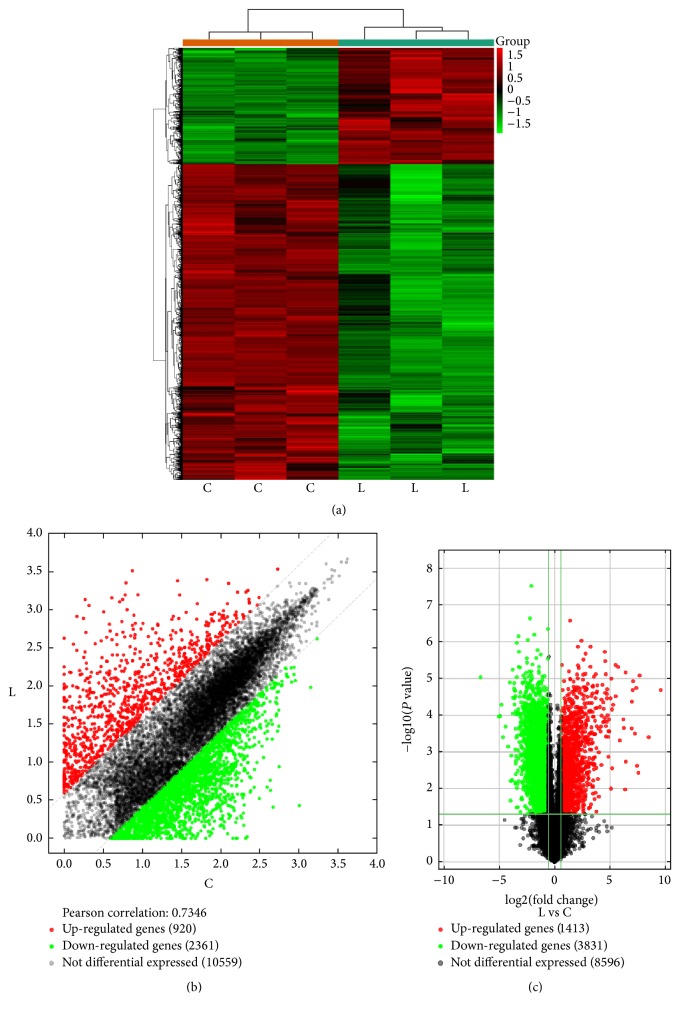
Differential expression of genes in lung tissues (fold change > 1.5, and *P* value < 0.05). (a) Hierarchical clustering analysis of genes that were differentially expressed in lung tissue samples between LPS-induced ARDS (group-L) and normal rat (group-C); each group contains 3 individuals. Green-black indicates lower expression, and red indicates high expression. (b) The scatter plot is used for evaluating the changing expression profiles of genes between group-L and group-C tissue samples. The values corresponding to the *x*- and *y*-axes in the scatter plot are the normalized signal values of the samples (log⁡2 scaled). The diagonal dotted lines represent fold changes. The genes above the top green line and below the bottom green line represent the differential expression genes. (c) The differentially expressed genes (shown in red font) with statistical significance from lung tissues between group-C and group-L screened using a volcano plot. The vertical line represents a boundary of the differential and nondifferential expression genes, and the horizontal line corresponds to a *P* value equal to 0.05.

Hierarchical clustering, together with the scatter and volcano plots, revealed 5244 significantly DEGs (Figure 1). It is clear that such clusterization successfully separates “ARDS” samples from “normal” ones, without any outlier. All 6 samples were included for further analysis.

### 3.4. Identification and Analysis of DEGs

Illumina HiSeq 4000 was used to investigate DEGs in the lung tissues from rats in both groups. Transcriptome data were generated and RNA-Seq reads of lung tissues were acquired. It was observed that, on an average, 97.98% (control group) and 99.99% (ARDS group) sequence reads passed the quality control (Table S2). Then these sequences were mapped successfully onto the rat genome RN6 using Hisat2 (v2.0.4). These reads were further subjected to annotate with the putative transcripts, quantification of the number of reads per transcript, and statistical comparison of transcript abundance across samples.

A total of 5244 genes were considered to be significant, with the *P* value < 0.05 and FC > 1.5. Of these genes, 1413 genes were upregulated and 3831 downregulated (Supplemental Excel 1). We ranked the genes according to FC expression levels and listed the top 11 significantly upregulated candidates of genes (FC > 100) in Table 1.

### 3.5. GO Analysis of the Biological Function of DEGs

**Figure 2:**
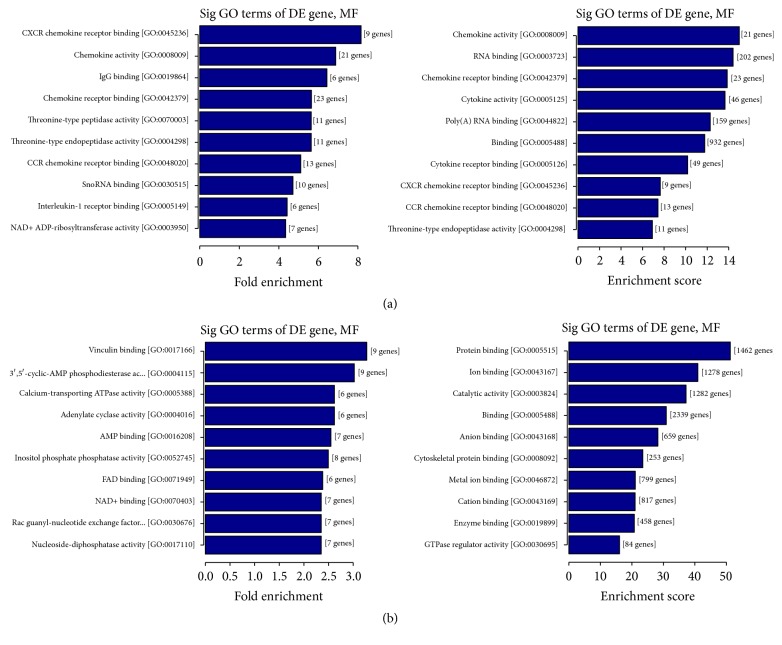
GO analysis of the biological function of differentially regulated genes. (a) The upregulated GO MF terms for the genes were analyzed. Top 10 upregulated GO terms ranked by fold enrichment and enrichment score were shown. (b) The downregulated GO MF terms for the genes were analyzed. Top 10 downregulated GO terms ranked by fold enrichment and enrichment score were shown.

GO analysis (http://www.geneontology.org/) was applied to search for significantly enriched GO terms which were made on biological process (BP), cellular component (CC), and molecular function (MF) for DEGs. Prediction terms with *P* value less than 0.05 were selected and ranked by fold enrichment ((Count/Pop. Hits)/(List. Total/Pop. Total)) or enrichment score (−log⁡10(*P*-value)). According to the results, 1810 BP terms, 91 CC terms, and 147 MF terms were found upregulated in LPS group compared with control group. In contrast, 1466 BP terms, 240 CC terms, and 293 MF terms were found downregulated. Top 10 generally changed GO terms in LPS group classified by BP, CC, MF, and ranked by fold enrichment or enrichment score were listed in Figure 2 and Figure S2 (*P* < 1.0 × 10^−7^ in all 10 terms).

Almost all of the most enriched and meaningful BP terms were related to immune-inflammation response, for instance, “Fc-gamma receptor signaling pathway (GO:0038094),” “response to interferon-beta (GO:0035456),” “cellular response to interferon-beta (GO:0035458),” “Toll-like receptor 2 signaling pathway (GO:00034134),” “immune response (GO:0006955),” “immune response process (GO:0002376),” “response to other organism (GO:0051707),” and “innate immune response (GO:0045087).”

The most enriched CC terms were primarily about cell such as “nucleolus (GO:0005730),” “membrane-bounded organelle (GO:0043227),” “organelle (GO:0043226),” “preribosome (GO:0030684),” and “small-subunit processome (GO:0032040).”

As for GO MF terms ranked by either fold enrichment or enrichment score, the mainly enriched terms were closely related to chemokines and cytokines. Represented terms were “CXCR chemokine receptor binding (GO:0045236),” “chemokine activity (GO:0008009),” “chemokine receptor binding (GO:0042379),” “CCR chemokine receptor binding (GO:0048020),” “cytokine activity (GO:0005125),” and “interleukin-1 receptor binding (GO:0003950).”

Moreover, KEGG pathway analysis was made, and pathways (*P* < 0.05) were selected and ranked by gene counts. Overall, 5244 DEGs were involved in 135 KEGG pathways. Top 20 pathways were listed for up- and downregulated genes, respectively (Tables S3 and S4). KEGG pathway analysis showed that the upregulated genes were mainly enriched in pathways like cytokine-cytokine receptor interaction, cytosolic DNA-sensing pathway, and Jak-STAT signaling pathway. The downregulated genes were primarily enriched in pathways containing focal adhesion, valine, leucine and isoleucine degradation, and phosphatidylinositol signaling system.

### 3.6. Validation of RNA-Seq Results

qRT-PCR was used to verify the expression levels of the DEGs. Only genes upregulated in lung tissues with significant expression change of FC > 100 and *P* < 0.05 were selected, including CXCL1, 2, 6, 9, 10, and 11, CCL2 and 7, Mt2A, AC128848.1, and Orm1. The primers used for the qRT-PCR analysis for these 11 genes and the product length were shown in Table S1.

**Figure 3:**
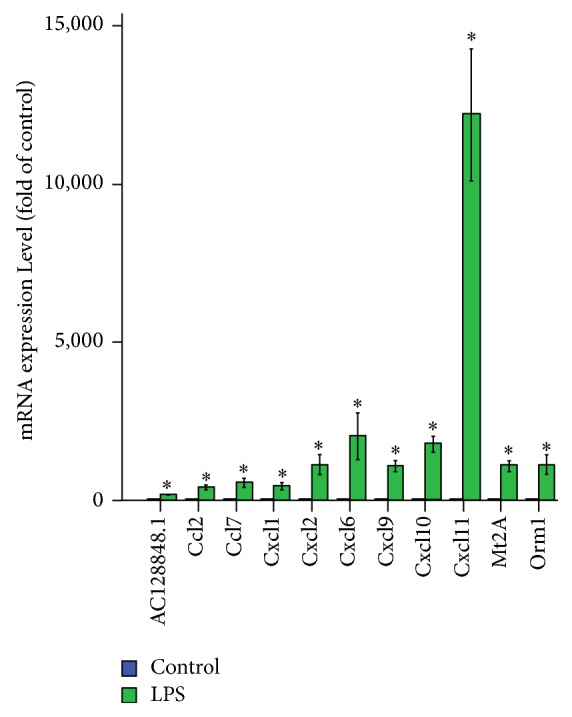
Eleven differentially expressed genes validated using qRT-PCR in rats with LPS-induced ARDS compared with normal rats. The data are presented as the mean ± SEM (*n* = 3) and analyzed by one-way analysis of variance followed by LSD posttest for multiple comparisons. ^*∗*^*P* < 0.01 versus control group.

The expression tendency of qRT-PCR was highly consistent with that of RNA-Seq when the two methods were compared and all 11 overexpressed genes exhibited significant validation. The expressions of all 11 genes were upregulated from 183- to 12183-fold (all *P* < 0.001) in LPS group versus control group (Figure 3). And the fold of LPS group relative to control group for CXCL11, CXCL9, Mt2A, AC128848.1, CXCL2, CCL2, CXCL10, CCL7, CXCL6, CXCL1, and Orm1 is 12183, 1100, 1107, 183, 1140, 428, 1799, 585, 2032, 473, and 1144, respectively.

In order to define how these 11 most upregulated genes interact with each other, we identified potential networks for these DEGs. Signal-net analysis integrated these 11 genes using STRING analysis and sixty nodes were involved in the establishment of gene regulation network, with 1251 edges, as depicted in Figure S3.

## 4. Discussion

ARDS remains a life-threatening lung disease and is associated with high in-hospital mortality of approximately 40% despite advances in critical care [24]. Currently, there is no Food and Drug Administration-approved effective pharmacologic treatment [25]. Furthermore, few biomarkers can be used to predict the initiation and progression of ARDS, to evaluate the response to treatment, to stratify the risk factors, or to predict prognosis [26]. The identification of more meaningful biomarkers and novel signaling pathways is required to provide new insights into the exact pathogenesis of the disease and to identify new therapeutic targets. Therefore, the regulation of mRNAs in the pathogenesis of ARDS needs further investigation.

Next-generation RNA-Seq is one of the preferred approaches to characterize and quantify the entire genome [27]. It furnishes a far more exact computation of transcript levels than any other techniques. Therefore, next-generation RNA-Seq lays a path for the detection of genes that have low expression levels [28]. Using RNA-Seq analysis, we identified 5244 DEGs in the lung tissues from LPS-treated rats compared with normal rats. Of these genes, roughly 1400 were upregulated and 3800 downregulated.

The present study highlights the ability of RNA-Seq analysis to detect DEGs in a rat model of ARDS induced by LPS. And these DEGs are expected to play specific roles in the pathogenesis of ARDS and maybe serve as useful biomarkers and potential therapeutic targets.

We chose 11 genes with expression levels that were significantly upregulated for further verification by qRT-PCR. Among the most upregulated 11 genes, there were 8 chemokine genes, including CXC (CXCL1, 2, 6, 9, 10, and 11) and CC (CCL2 and 7) family members. The expression tendency of all 11 genes qRT-PCR was highly consistent with that of RNA-Seq. We found that almost all of the most enriched BP terms for DEGs were related to immune-inflammation response. We also found that, as for GO MF terms, the enriched terms were mainly related to chemokines and cytokines. Our findings agree with increasing evidence that the immune system and intense pulmonary inflammation play key roles in ARDS and highlight the important effect of chemokines on the process of inflammation response [29].

The innate immune response acts as a potent driving force for ARDS [30]. When resident alveolar macrophages are stimulated by pathogen recognition, much of the production of the early cytokines, mainly IL-1*β* and TNF-*α*, is released. These cytokines in turn stimulate neighbouring cells to produce a battery of chemokines. These chemokines direct an influx of excessive neutrophils and other inflammatory cells and activation of endothelial cell resulting in increased vascular permeability [30–34]. Pulmonary neutrophils then release defensive mediators that can lead to further damage in the process of ARDS.

The local chemokines orchestrating the recruitment of neutrophils into the lung include CCL2 and 7 and CXCL1, 2, 6, and 10. [31, 35–41]. Apart from being as a potent chemotactic factor for neutrophils, CCL2 is also responsible for over 95% of the monocyte chemotactic activity produced by alveolar macrophages from acutely injured rat lungs [42]. In humans, CCL7 activates dendritic cells, eosinophils, and basophils with being the only chemokine to induce the migration of M1 and M2 macrophages [43, 44]. CXCL9 is a T-cell chemoattractant and is closely related to CXCL10 and 11 [45]. CXCL10 appears to be a pivotal factor for the exacerbation of the inflammatory response [46]. Furthermore, chemokines have a particularly profound effect on lung fluid balance since they can alter both barrier function and alveolar amiloride-sensitive epithelial sodium channels [2].

Several reports have demonstrated that CXCL1, CXCL2, and JE (the murine homolog of human CCL2) accumulated in bronchoalveolar lavage fluid from mice with HCl- or LPS-induced ARDS, CCL2 in bleomycin-injured mouse lungs, CXCL2 and 9 in LPS-treated mouse serum and lung, and mRNA expression of CCL2 in lungs from repeated saline lavage-induced and mechanical ventilation-induced rats, CCL2 and 7 in bronchoalveolar lavage fluid from LPS-challenged volunteers and ARDS patients, and the mRNA and protein expression of CCL2 in LPS-treated RAW264.7 cells [31, 47–53]. As mentioned in a recent study, CCL2 and 7 and CXCL1, 10, and 11 showed significantly different expression levels in lung tissues from LPS-induced ARDS mice by digital gene expression analysis [54]. In a previous study, the production of CCL2 was blocked and thereby decreased the influx of inflammatory cells during lung injury [53]. Neutralizing either CCL2 or 7 attenuated the neutrophil chemotactic response [31]. However, CCL2 transgenic mice expressing human CCL2 have increased numbers of lymphocytes and monocytes in the airway [55]. Neutralization of CXCL10 ameliorates the severity of ARDS by inhibiting inflammatory cells recruitment into the lung, decreasing the production of inflammatory mediators, and consequently reducing pulmonary edema [56]. Therefore, in the process of ARDS, chemokines may have a considerable regulatory function in inflammation process and immune response. All these studies agree with our present study suggesting lots of upregulated chemokine genes in lung tissues that were harvested after the rats were treated with LPS.

We found a significant upregulated metallothionein 2A gene expression that represented the enhanced inflammation response [57]. In two studies by Perkowski et al. and Lingappan et al., metallothionein was among the top three upregulated genes in 8–10-week-old female mice after hyperoxia exposure for 48 h [58, 59]. Karthikeyan and coworkers revealed that, in the rat lungs, single exposures to diesel exhaust resulted in increased metallothionein 2A gene expression [57]. In a recent study of rats with repeated saline lavage-induced and mechanical ventilation-induced ARDS conducted by Huang C, the mRNA expression of metallothionein 2A in lungs also showed a twofold increase. Metallothionein was reported to scavenge hydroxyl radicals and acted as an antioxidant [60]. Metallothionein 2A had been shown to interact with protein kinase D1 which functioned in many extracellular receptor-mediated signal transduction pathways [61]. AC128848.1 is also a metallothionein, but its exact function is not clear.

Orm1 encoded a key acute phase plasma protein due to acute inflammation, involved in aspects of immunosuppression [62]. In reviewing literature, no data was found on the association between ARDS and the change in the Orm1 gene expression.

Consideration chemokines as biomarkers for ARDS have provided valuable insight into the pathogenesis. This is a new hope of identifying new biomarkers for prediction, prognostication, and diagnosis of ARDS. Neutralization of upregulated chemokines by mRNA antagonists can restrain the development of ARDS [31, 53, 56]. Our present study and these previous studies suggest that chemokines can act as therapeutic targets for ARDS cases of different etiologies.

However, one of the limitations of this study is that the significant increase of chemokines we validated was in lungs from LPS-induced ARDS rats. We need to design a further study to investigate if these changes also exist in plasma and bronchoalveolar lavage fluid from an ARDS animal model. Meanwhile, our study might have been more meaningful if we expand the present study to human cell lines, tissues, and subjects because it would provide direct evidence to the role of mRNAs in the development of ARDS in human.

## 5. Conclusion

In summary, we revealed some DEGs in lung tissue samples from ARDS rats that might play important roles in the pathogenesis of ARDS and found that a lot of chemokine family members were among the most upregulated genes. Almost all of the most enriched and meaningful BP terms were related to immune-inflammation response. We also revealed that, as for GO MF terms, the enriched terms were mainly related to chemokines and cytokines. Further well-designed studies exploring the roles of these chemokine family members in the pathogenesis of ARDS and developing diagnostic panels and therapeutic targets based on these aberrantly expressed chemokines are needed.

## Figures and Tables

**Table 1:** The top 11 significantly upregulated genes ranking by fold change (>100) in LPS group versus control group.

Rank	Gene name	Fold change	*P*
(1)	Cxcl11	770.5755395	2.06684*E* − 05
(2)	Cxcl9	361.1128287	0.00039737
(3)	Mt2A	206.1296149	8.31066*E* − 06
(4)	AC128848.1	190.6579384	0.003713725
(5)	Cxcl2	174.3016701	0.002376845
(6)	Ccl2	168.2184472	1.83426*E* − 05
(7)	Cxcl10	152.3927108	0.000315332
(8)	Ccl7	137.7664371	1.19045*E* − 05
(9)	Cxcl6	134.414233	2.07021*E* − 05
(10)	Cxcl1	126.8603041	4.28187*E* − 05
(11)	Orm1	125.1164755	0.000228345

## Supplementary Materials

Supplementary Materials Figure S1: LPS-induced ARDS in rats. Sprague-Dawley rats were treated with LPS (20 mg/kg) via caudal vein 7 h before the lung tissues were collected, and the effect of LPS was assessed by histology in H&E-stained sections (bars = 100 *μ*m; original magnification ×400). Figure S2: GO analysis of the biological function of genes. (A-B) Upregulated GO BP and CC terms for the DEGs were analyzed. Top 10 upregulated GO terms ranked by fold enrichment and enrichment score were shown. (C-D) Downregulated GO BP and CC terms for the DEGs were analyzed. Top 10 downregulated GO terms ranked by fold enrichment and enrichment score were shown. Figure S3: STRING analysis of interaction in the altered genes in rat ARDS. Nodes represent genes. Genes with more links are shown in a bigger size. Purple lines represent experimental evidence; yellow lines represent text-mining evidence; light lines represent database evidence. Table S1: the detailed characteristics of qRT-PCR for 11 candidate genes. Table S2: RNA-Seq reads and mapping rate of lung tissues from ARDS rats and normal rats. Table S3: upregulated KEGG pathway analysis. Table S4: downregulated KEGG pathway analysis. Supplemental Excel 1: a total of 5244 genes were considered to be significant, with the *P* value of <0.05 and FC > 1.5. Of those genes, 1413 genes were upregulated and 3831 downregulated.
