# Supplementary material for: Candidate Genes as Biomarkers in Lipopolysaccharide-Induced Acute Respiratory Distress Syndrome Based on mRNA Expression Profile by Next-Generation RNA-Seq Analysis
Source: Biomed Res Int. 2018 Apr 8;2018:4384797. doi: 10.1155/2018/4384797 (PMC5911337; doi:10.1155/2018/4384797)
Supplement: Supplementary Materials — Figure S1: LPS-induced ARDS in rats. Sprague-Dawley rats were treated with LPS (20 mg/kg) via caudal vein 7 h before the lung tissues were collected, and the effect of LPS was assessed by histology in H&E-stained sections (bars = 100 μm; original magnification ×400). Figure S2: GO analysis of the biological function of genes. (A-B) Upregulated GO BP and CC terms for the DEGs were analyzed. Top 10 upregulated GO terms ranked by fold enrichment and enrichment score were shown. (C-D) Downregulated GO BP and CC terms for the DEGs were analyzed. Top 10 downregulated GO terms ranked by fold enrichment and enrichment score were shown. Figure S3: STRING analysis of interaction in the altered genes in rat ARDS. Nodes represent genes. Genes with more links are shown in a bigger size. Purple lines represent experimental evidence; yellow lines represent text-mining evidence; light lines represent database evidence. Table S1: the detailed characteristics of qRT-PCR for 11 candidate genes. Table S2: RNA-Seq reads and mapping rate of lung tissues from ARDS rats and normal rats. Table S3: upregulated KEGG pathway analysis. Table S4: downregulated KEGG pathway analysis. Supplemental Excel 1: a total of 5244 genes were considered to be significant, with the P value of <0.05 and FC > 1.5. Of those genes, 1413 genes were upregulated and 3831 downregulated. [file 4384797.f1.zip › 4384797.f1/Table S_BMRI_2158925.docx]

Table S1.The detailed characteristics of qRT-PCR for 11 candidate genes

| Target name | Primer sequence | Annealing Temperature (℃) | Product length (bp) |
| --- | --- | --- | --- |
| β-actin（R） | F:5'CGAGTACAACCTTCTTGCAGC3’  R :5’ ACCCATACCCACCATCACAC3’ | 60 | 202 |
| Cxcl11 | F:5'AGATGAACAGGACGGGCAT 3’  R :5’ GCTGCCATTTTGACCACTTTC 3’ | 60 | 133 |
| Cxcl9 | F:5'CACATTCCACTACAAATCCCT 3’  R :5’ TCTCCCATTCTTTCATCAGCT 3’ | 60 | 155 |
| Mt2A | F:5'TAAAACAACGTAGGAACCTAG 3’  R :5’ TCAAGTCAAGTTTTTTTTATTT 3’ | 60 | 108 |
| AC128848.1 | F:5'AGGAAGCCTACTCTGACATCTTCC 3’  R :5’ GAGCAGCAGCCTGGAAAGAAA 3’ | 60 | 50 |
| Cxcl2 | F:5' CAATGCTGTACTGGTCCTGCTC 3’  R :5’ CAGTTAGCCTTGCCTTTGTTCA 3’ | 60 | 276 |
| Ccl2 | F:5'GCTACTCATTCACTGGCAAGA 3’  R :5’ CTTATTGGGGTCAGCACAGAT 3’ | 60 | 137 |
| Cxcl10 | F:5'AGCCAACCTTCCAGAAGCACCA 3’  R :5’ TCATGGAAGTCGATGCAGGTGC 3’ | 60 | 131 |
| Ccl7 | F:5'CGCTTCTGTGTGTGCTGCTCA 3’  R :5’GCCTCCTCAACCCACTTCTG 3’ | 60 | 235 |
| Cxcl6 | F:5'GTTTGCTTAACCTTAGCTCCA 3’  R :5’ GTTTTCTTATTTTCACTGCCC 3’ | 60 | 197 |
| Cxcl1 | F:5' GTGTTTTGTGTTAGGGTGAGG 3’  R :5’ GACGAGAAGGAGCATTGGTTA 3’ | 60 | 189 |
| Orm1 | F:5'ACTTCGGGAGTTTCAGACCAC 3’  R :5’ CTTCAGCACTATCAAATGGGC 3’ | 60 | 133 |

Table S2. RNA-seq reads and mapping rate of lung tissues from ARDS and normal rats

|  | C10 | C8 | C9 | L2 | L3 | L5 |
| --- | --- | --- | --- | --- | --- | --- |
| Raw Pairs | 24,862,137 | 25,075,706 | 23,903,618 | 23,354,088 | 16,694,337 | 20,361,198 |
| Trimmed Pairs | 24,859,876  (99.99%) | 25,068,097  (99.97%) | 23,902,031  (99.99%) | 23,351,870  (99.99%) | 16,692,915  (99.99%) | 20,359,364  (99.99%) |
| Average accepted hit |  | 99.98% |  |  | 99.99% |  |

Table S3. Up-regulated KEGG pathway analysis

| Pathway ID | | Definition | Counts | | Enrichment_Score | *P*-value |
| --- | --- | --- | --- | --- | --- | --- |
| rno04060 | Cytokine-cytokine receptor interaction | | | 58 | 17.74946384 | 1.78048E-18 |
| rno04623 | Cytosolic DNA-sensing pathway | | | 21 | 11.15851148 | 6.94206E-12 |
| rno04630 | Jak-STAT signaling pathway | | | 36 | 9.399436997 | 3.98624E-10 |
| rno04621 | NOD-like receptor signaling pathway | | | 20 | 8.809221932 | 1.55159E-09 |
| rno04380 | Osteoclast differentiation | | | 31 | 8.49139645 | 3.22555E-09 |
| rno04210 | Apoptosis | | | 26 | 8.150423076 | 7.07256E-09 |
| rno03050 | Proteasome | | | 18 | 7.614789861 | 2.42778E-08 |
| rno04620 | Toll-like receptor signaling pathway | | | 25 | 7.586975166 | 2.58836E-08 |
| rno03008 | Ribosome biogenesis in eukaryotes | | | 23 | 7.232190488 | 5.85881E-08 |
| rno05140 | Leishmaniasis | | | 20 | 6.778777463 | 1.66427E-07 |
| rno04622 | RIG-I-like receptor signaling pathway | | | 19 | 6.678877516 | 2.0947E-07 |
| rno04062 | Chemokine signaling pathway | | | 35 | 6.196880785 | 6.35505E-07 |
| rno05160 | Hepatitis C | | | 26 | 5.180882132 | 6.59353E-06 |
| rno04640 | Hematopoietic cell lineage | | | 19 | 4.99083978 | 1.02132E-05 |
| rno05145 | Toxoplasmosis | | | 25 | 4.446383026 | 3.57781E-05 |
| rno05142 | Chagas disease (American trypanosomiasis) | | | 21 | 4.304451082 | 4.96077E-05 |
| rno04145 | Phagosome | | | 31 | 4.149344009 | 7.09016E-05 |
| rno05143 | African trypanosomiasis | | | 11 | 4.138672724 | 7.26653E-05 |
| rno05332 | Graft-versus-host disease | | | 15 | 4.129223489 | 7.42637E-05 |
| rno05144 | Malaria | | | 14 | 3.8906942 | 0.000128619 |

Table S4. Down-regulated KEGG pathway analysis

| Pathway ID | | Definition | Counts | | Enrichment_Score | *P*-value |
| --- | --- | --- | --- | --- | --- | --- |
| rno04510 | Focal adhesion | | | 75 | 13.65620322 | 2.20697E-14 |
| rno00280 | Valine, leucine and isoleucine degradation | | | 29 | 10.40267862 | 3.95659E-11 |
| rno04070 | Phosphatidylinositol signaling system | | | 36 | 9.033468949 | 9.2583E-10 |
| rno01100 | Metabolic pathways | | | 265 | 8.090975479 | 8.11007E-09 |
| rno04270 | Vascular smooth muscle contraction | | | 44 | 7.198547771 | 6.33071E-08 |
| rno04512 | ECM-receptor interaction | | | 32 | 6.920831146 | 1.19997E-07 |
| rno05200 | Pathways in cancer | | | 89 | 6.636708723 | 2.30829E-07 |
| rno04916 | Melanogenesis | | | 37 | 6.485664966 | 3.2684E-07 |
| rno04360 | Axon guidance | | | 44 | 6.014687449 | 9.66746E-07 |
| rno04810 | Regulation of actin cytoskeleton | | | 63 | 5.875354735 | 1.33243E-06 |
| rno00310 | Lysine degradation | | | 22 | 5.838017567 | 1.45205E-06 |
| rno00340 | Histidine metabolism | | | 15 | 5.744575004 | 1.80063E-06 |
| rno00640 | Propanoate metabolism | | | 18 | 5.687153835 | 2.05516E-06 |
| rno04970 | Salivary secretion | | | 30 | 5.513925705 | 3.06249E-06 |
| rno04142 | Lysosome | | | 42 | 5.479259435 | 3.31696E-06 |
| rno05217 | Basal cell carcinoma | | | 23 | 5.343177714 | 4.53756E-06 |
| rno00562 | Inositol phosphate metabolism | | | 25 | 5.342611629 | 4.54348E-06 |
| rno00071 | Fatty acid degradation | | | 21 | 5.194000205 | 6.39735E-06 |
| rno00230 | Purine metabolism | | | 50 | 4.885252664 | 1.30241E-05 |
| rno05100 | Bacterial invasion of epithelial cells | | | 27 | 4.803555092 | 1.57197E-05 |


