# Supplementary figures and images for: Candidate Genes as Biomarkers in Lipopolysaccharide-Induced Acute Respiratory Distress Syndrome Based on mRNA Expression Profile by Next-Generation RNA-Seq Analysis
Source: Biomed Res Int. 2018 Apr 8;2018:4384797. doi: 10.1155/2018/4384797 (PMC5911337; doi:10.1155/2018/4384797)

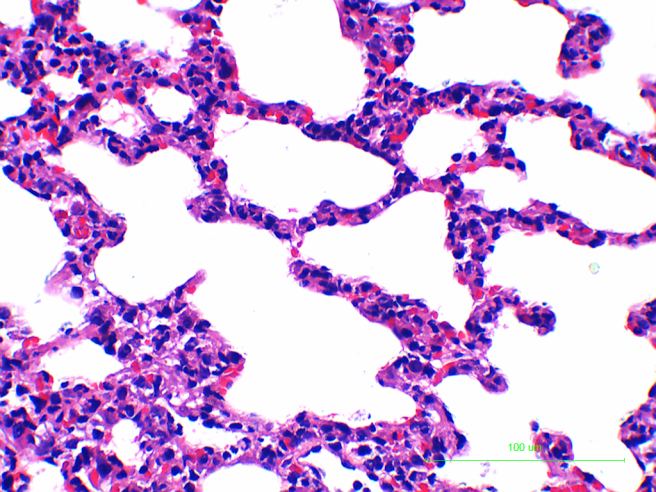

Supplement: Supplementary Materials — Figure S1: LPS-induced ARDS in rats. Sprague-Dawley rats were treated with LPS (20 mg/kg) via caudal vein 7 h before the lung tissues were collected, and the effect of LPS was assessed by histology in H&E-stained sections (bars = 100 μm; original magnification ×400). Figure S2: GO analysis of the biological function of genes. (A-B) Upregulated GO BP and CC terms for the DEGs were analyzed. Top 10 upregulated GO terms ranked by fold enrichment and enrichment score were shown. (C-D) Downregulated GO BP and CC terms for the DEGs were analyzed. Top 10 downregulated GO terms ranked by fold enrichment and enrichment score were shown. Figure S3: STRING analysis of interaction in the altered genes in rat ARDS. Nodes represent genes. Genes with more links are shown in a bigger size. Purple lines represent experimental evidence; yellow lines represent text-mining evidence; light lines represent database evidence. Table S1: the detailed characteristics of qRT-PCR for 11 candidate genes. Table S2: RNA-Seq reads and mapping rate of lung tissues from ARDS rats and normal rats. Table S3: upregulated KEGG pathway analysis. Table S4: downregulated KEGG pathway analysis. Supplemental Excel 1: a total of 5244 genes were considered to be significant, with the P value of <0.05 and FC > 1.5. Of those genes, 1413 genes were upregulated and 3831 downregulated. [file 4384797.f1.zip › 4384797.f1/Figure S1-Control_BMRI_2212873.pdf]

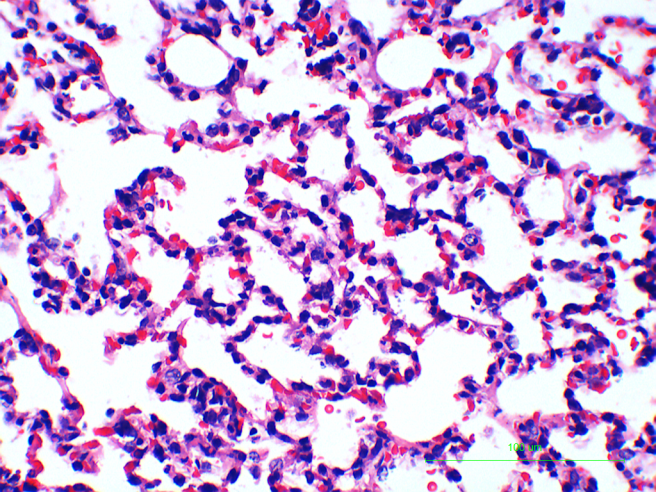

100 µm

Supplement: Supplementary Materials — Figure S1: LPS-induced ARDS in rats. Sprague-Dawley rats were treated with LPS (20 mg/kg) via caudal vein 7 h before the lung tissues were collected, and the effect of LPS was assessed by histology in H&E-stained sections (bars = 100 μm; original magnification ×400). Figure S2: GO analysis of the biological function of genes. (A-B) Upregulated GO BP and CC terms for the DEGs were analyzed. Top 10 upregulated GO terms ranked by fold enrichment and enrichment score were shown. (C-D) Downregulated GO BP and CC terms for the DEGs were analyzed. Top 10 downregulated GO terms ranked by fold enrichment and enrichment score were shown. Figure S3: STRING analysis of interaction in the altered genes in rat ARDS. Nodes represent genes. Genes with more links are shown in a bigger size. Purple lines represent experimental evidence; yellow lines represent text-mining evidence; light lines represent database evidence. Table S1: the detailed characteristics of qRT-PCR for 11 candidate genes. Table S2: RNA-Seq reads and mapping rate of lung tissues from ARDS rats and normal rats. Table S3: upregulated KEGG pathway analysis. Table S4: downregulated KEGG pathway analysis. Supplemental Excel 1: a total of 5244 genes were considered to be significant, with the P value of <0.05 and FC > 1.5. Of those genes, 1413 genes were upregulated and 3831 downregulated. [file 4384797.f1.zip › 4384797.f1/Figure S1-LPS_BMRI_2212874.pdf]

# Sig GO terms of DE gene–BP

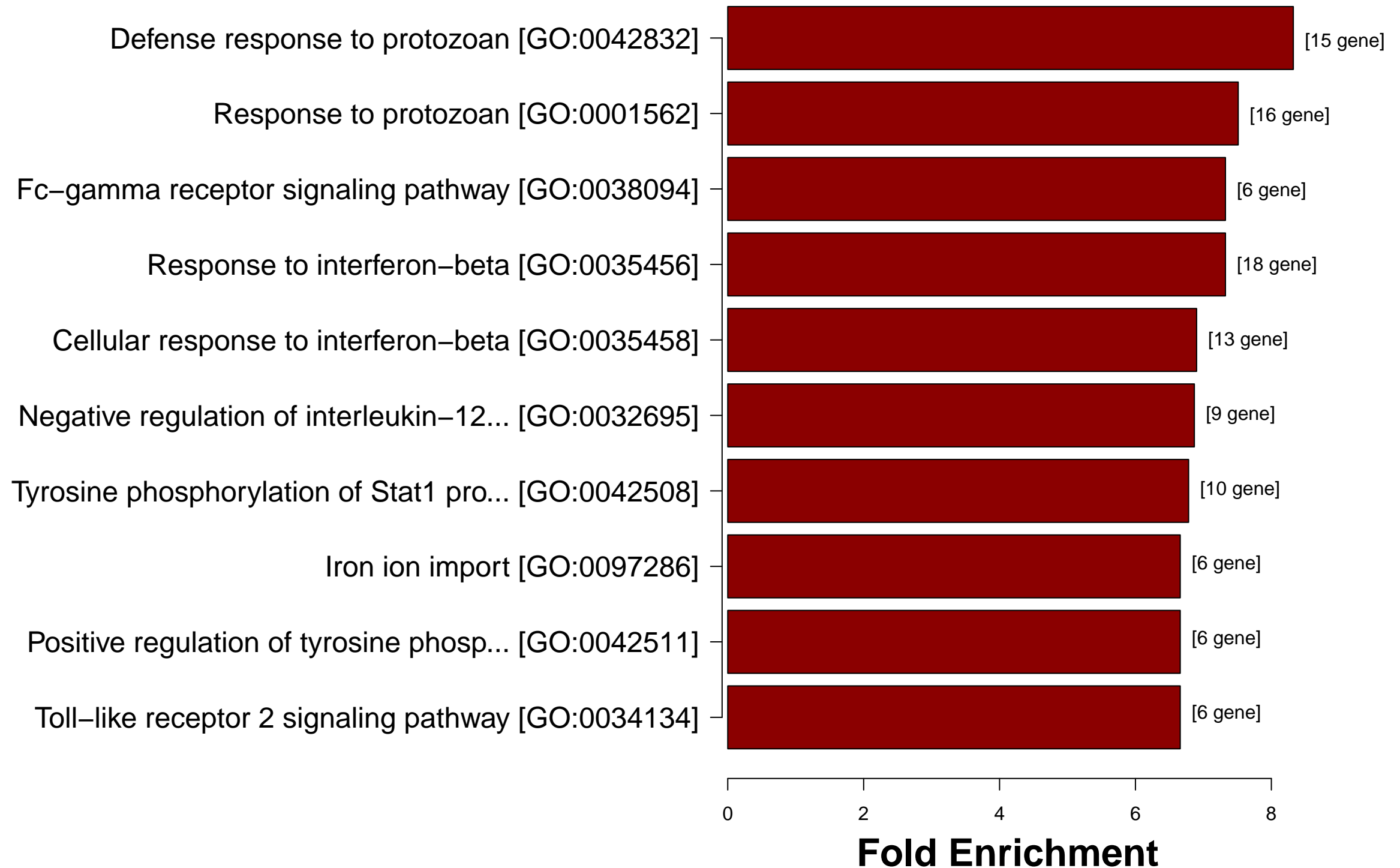

Supplement: Supplementary Materials — Figure S1: LPS-induced ARDS in rats. Sprague-Dawley rats were treated with LPS (20 mg/kg) via caudal vein 7 h before the lung tissues were collected, and the effect of LPS was assessed by histology in H&E-stained sections (bars = 100 μm; original magnification ×400). Figure S2: GO analysis of the biological function of genes. (A-B) Upregulated GO BP and CC terms for the DEGs were analyzed. Top 10 upregulated GO terms ranked by fold enrichment and enrichment score were shown. (C-D) Downregulated GO BP and CC terms for the DEGs were analyzed. Top 10 downregulated GO terms ranked by fold enrichment and enrichment score were shown. Figure S3: STRING analysis of interaction in the altered genes in rat ARDS. Nodes represent genes. Genes with more links are shown in a bigger size. Purple lines represent experimental evidence; yellow lines represent text-mining evidence; light lines represent database evidence. Table S1: the detailed characteristics of qRT-PCR for 11 candidate genes. Table S2: RNA-Seq reads and mapping rate of lung tissues from ARDS rats and normal rats. Table S3: upregulated KEGG pathway analysis. Table S4: downregulated KEGG pathway analysis. Supplemental Excel 1: a total of 5244 genes were considered to be significant, with the P value of <0.05 and FC > 1.5. Of those genes, 1413 genes were upregulated and 3831 downregulated. [file 4384797.f1.zip › 4384797.f1/Figure S2A1_BMRI_2212876.pdf]

# Sig GO terms of DE gene-BP

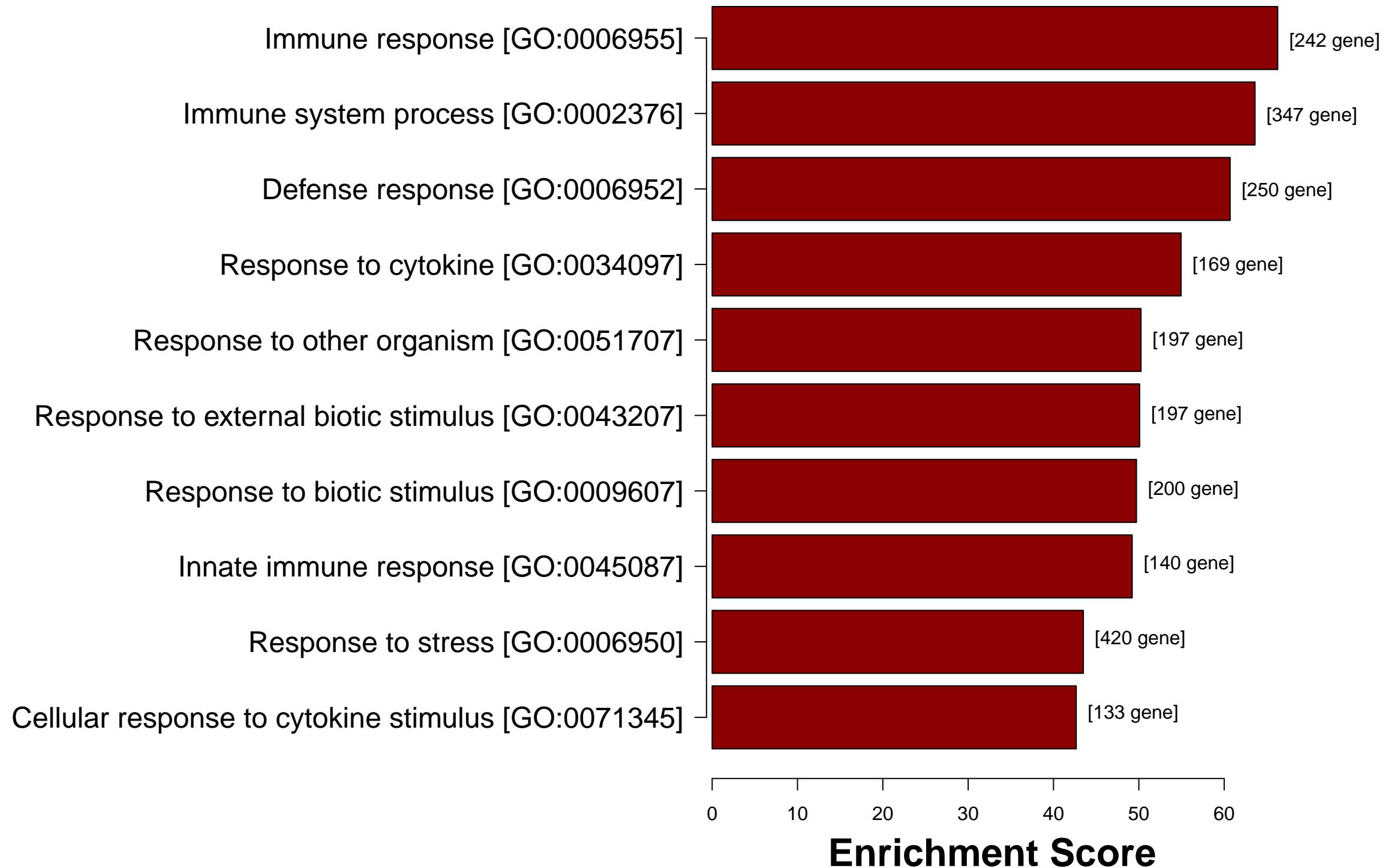

Supplement: Supplementary Materials — Figure S1: LPS-induced ARDS in rats. Sprague-Dawley rats were treated with LPS (20 mg/kg) via caudal vein 7 h before the lung tissues were collected, and the effect of LPS was assessed by histology in H&E-stained sections (bars = 100 μm; original magnification ×400). Figure S2: GO analysis of the biological function of genes. (A-B) Upregulated GO BP and CC terms for the DEGs were analyzed. Top 10 upregulated GO terms ranked by fold enrichment and enrichment score were shown. (C-D) Downregulated GO BP and CC terms for the DEGs were analyzed. Top 10 downregulated GO terms ranked by fold enrichment and enrichment score were shown. Figure S3: STRING analysis of interaction in the altered genes in rat ARDS. Nodes represent genes. Genes with more links are shown in a bigger size. Purple lines represent experimental evidence; yellow lines represent text-mining evidence; light lines represent database evidence. Table S1: the detailed characteristics of qRT-PCR for 11 candidate genes. Table S2: RNA-Seq reads and mapping rate of lung tissues from ARDS rats and normal rats. Table S3: upregulated KEGG pathway analysis. Table S4: downregulated KEGG pathway analysis. Supplemental Excel 1: a total of 5244 genes were considered to be significant, with the P value of <0.05 and FC > 1.5. Of those genes, 1413 genes were upregulated and 3831 downregulated. [file 4384797.f1.zip › 4384797.f1/Figure S2A2_BMRI_2212877.pdf]

# Sig GO terms of DE gene-CC

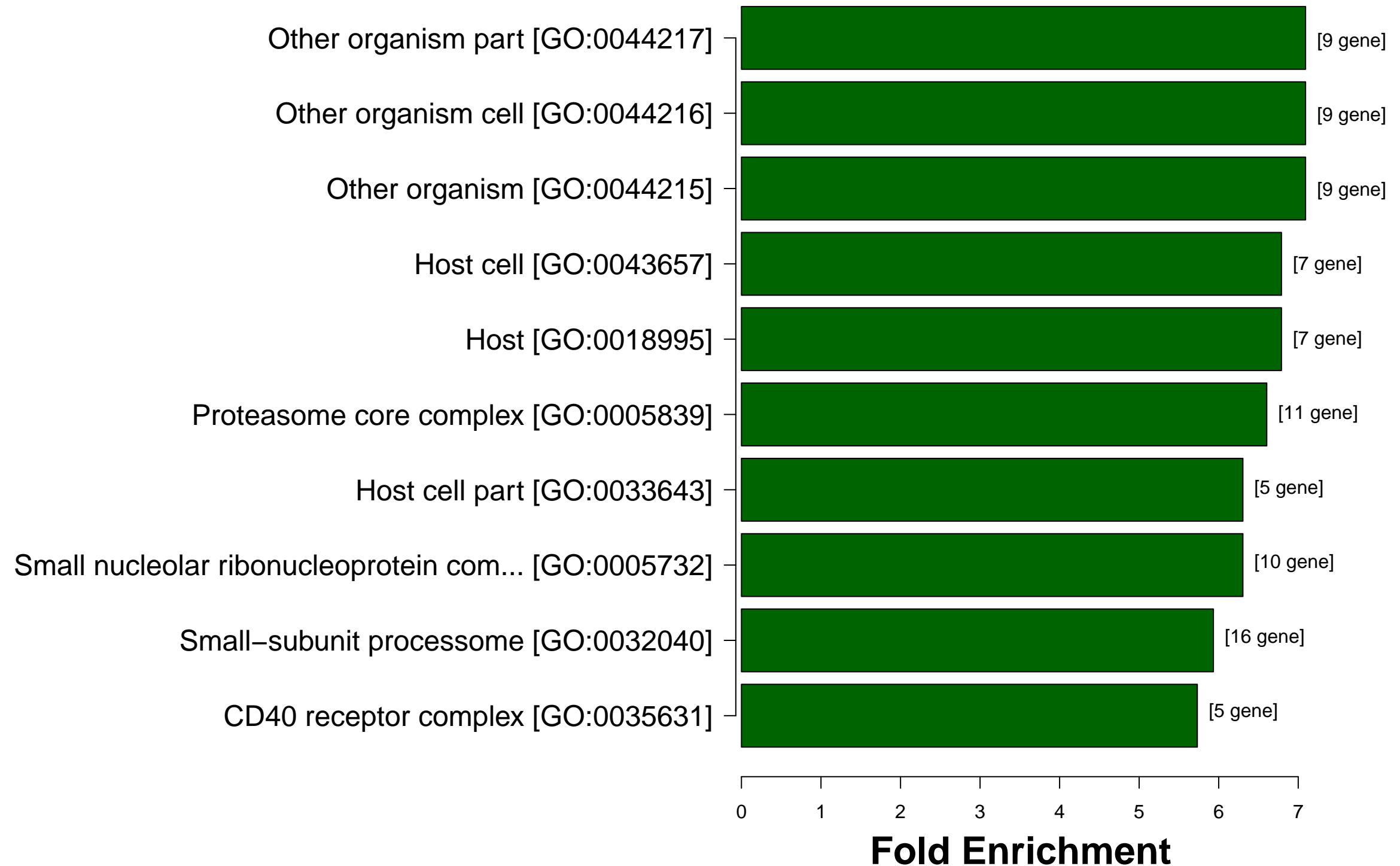

Supplement: Supplementary Materials — Figure S1: LPS-induced ARDS in rats. Sprague-Dawley rats were treated with LPS (20 mg/kg) via caudal vein 7 h before the lung tissues were collected, and the effect of LPS was assessed by histology in H&E-stained sections (bars = 100 μm; original magnification ×400). Figure S2: GO analysis of the biological function of genes. (A-B) Upregulated GO BP and CC terms for the DEGs were analyzed. Top 10 upregulated GO terms ranked by fold enrichment and enrichment score were shown. (C-D) Downregulated GO BP and CC terms for the DEGs were analyzed. Top 10 downregulated GO terms ranked by fold enrichment and enrichment score were shown. Figure S3: STRING analysis of interaction in the altered genes in rat ARDS. Nodes represent genes. Genes with more links are shown in a bigger size. Purple lines represent experimental evidence; yellow lines represent text-mining evidence; light lines represent database evidence. Table S1: the detailed characteristics of qRT-PCR for 11 candidate genes. Table S2: RNA-Seq reads and mapping rate of lung tissues from ARDS rats and normal rats. Table S3: upregulated KEGG pathway analysis. Table S4: downregulated KEGG pathway analysis. Supplemental Excel 1: a total of 5244 genes were considered to be significant, with the P value of <0.05 and FC > 1.5. Of those genes, 1413 genes were upregulated and 3831 downregulated. [file 4384797.f1.zip › 4384797.f1/Figure S2B1_BMRI_2212878.pdf]

# Sig GO terms of DE gene-CC

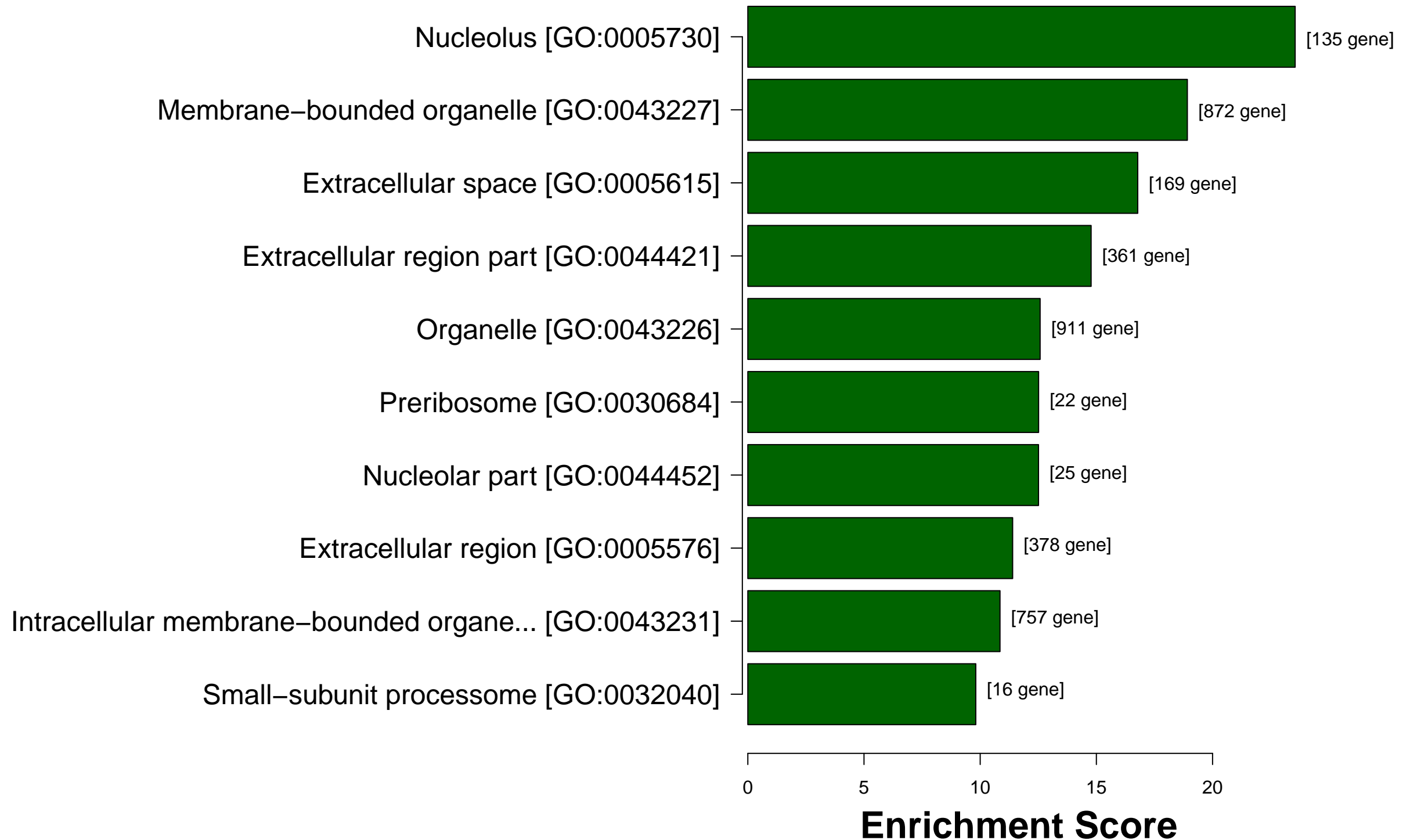

Supplement: Supplementary Materials — Figure S1: LPS-induced ARDS in rats. Sprague-Dawley rats were treated with LPS (20 mg/kg) via caudal vein 7 h before the lung tissues were collected, and the effect of LPS was assessed by histology in H&E-stained sections (bars = 100 μm; original magnification ×400). Figure S2: GO analysis of the biological function of genes. (A-B) Upregulated GO BP and CC terms for the DEGs were analyzed. Top 10 upregulated GO terms ranked by fold enrichment and enrichment score were shown. (C-D) Downregulated GO BP and CC terms for the DEGs were analyzed. Top 10 downregulated GO terms ranked by fold enrichment and enrichment score were shown. Figure S3: STRING analysis of interaction in the altered genes in rat ARDS. Nodes represent genes. Genes with more links are shown in a bigger size. Purple lines represent experimental evidence; yellow lines represent text-mining evidence; light lines represent database evidence. Table S1: the detailed characteristics of qRT-PCR for 11 candidate genes. Table S2: RNA-Seq reads and mapping rate of lung tissues from ARDS rats and normal rats. Table S3: upregulated KEGG pathway analysis. Table S4: downregulated KEGG pathway analysis. Supplemental Excel 1: a total of 5244 genes were considered to be significant, with the P value of <0.05 and FC > 1.5. Of those genes, 1413 genes were upregulated and 3831 downregulated. [file 4384797.f1.zip › 4384797.f1/Figure S2B2_BMRI_2212879.pdf]

# Sig GO terms of DE gene–BP

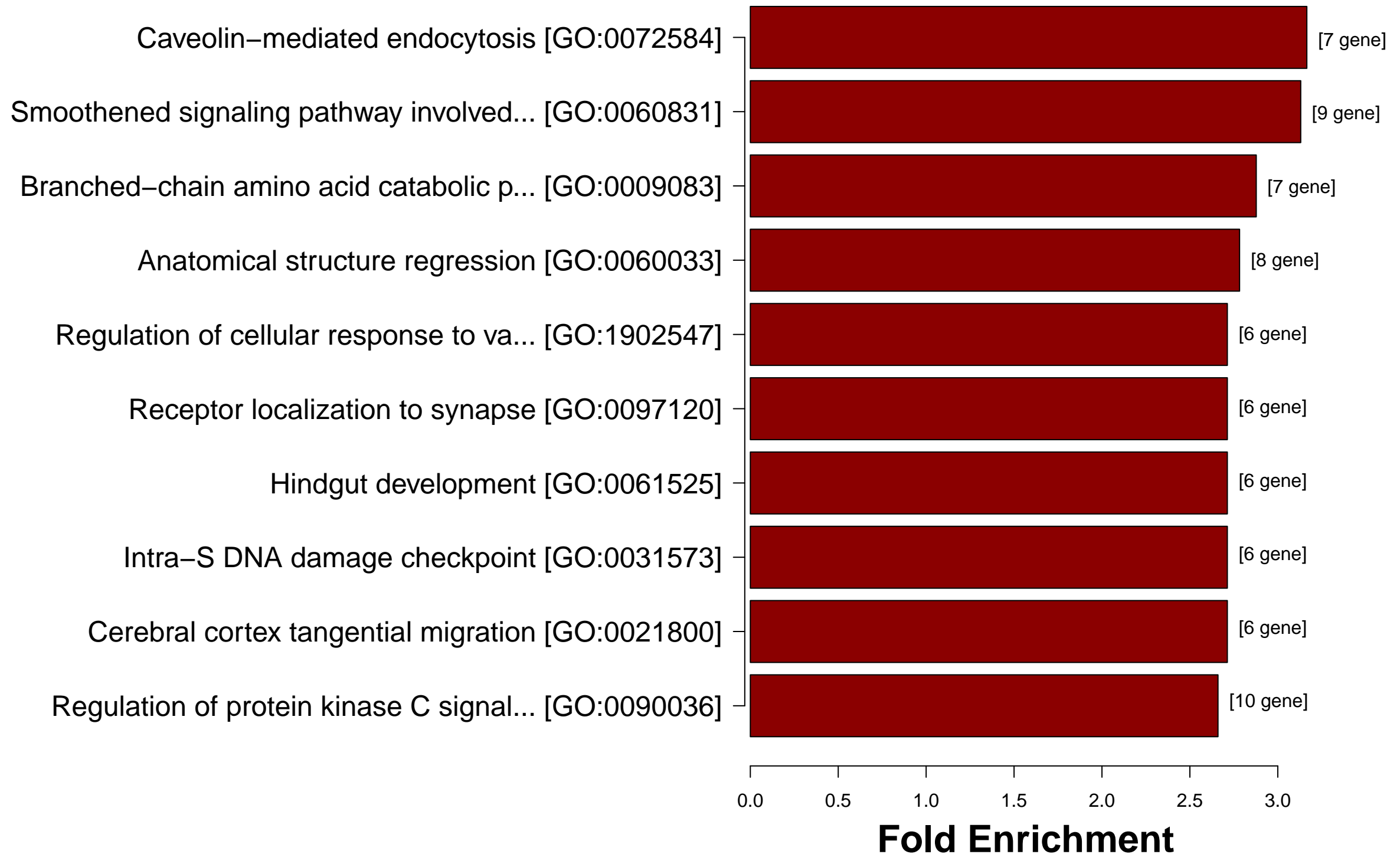

Supplement: Supplementary Materials — Figure S1: LPS-induced ARDS in rats. Sprague-Dawley rats were treated with LPS (20 mg/kg) via caudal vein 7 h before the lung tissues were collected, and the effect of LPS was assessed by histology in H&E-stained sections (bars = 100 μm; original magnification ×400). Figure S2: GO analysis of the biological function of genes. (A-B) Upregulated GO BP and CC terms for the DEGs were analyzed. Top 10 upregulated GO terms ranked by fold enrichment and enrichment score were shown. (C-D) Downregulated GO BP and CC terms for the DEGs were analyzed. Top 10 downregulated GO terms ranked by fold enrichment and enrichment score were shown. Figure S3: STRING analysis of interaction in the altered genes in rat ARDS. Nodes represent genes. Genes with more links are shown in a bigger size. Purple lines represent experimental evidence; yellow lines represent text-mining evidence; light lines represent database evidence. Table S1: the detailed characteristics of qRT-PCR for 11 candidate genes. Table S2: RNA-Seq reads and mapping rate of lung tissues from ARDS rats and normal rats. Table S3: upregulated KEGG pathway analysis. Table S4: downregulated KEGG pathway analysis. Supplemental Excel 1: a total of 5244 genes were considered to be significant, with the P value of <0.05 and FC > 1.5. Of those genes, 1413 genes were upregulated and 3831 downregulated. [file 4384797.f1.zip › 4384797.f1/Figure S2C1_BMRI_2212880.pdf]

# Sig GO terms of DE gene–BP

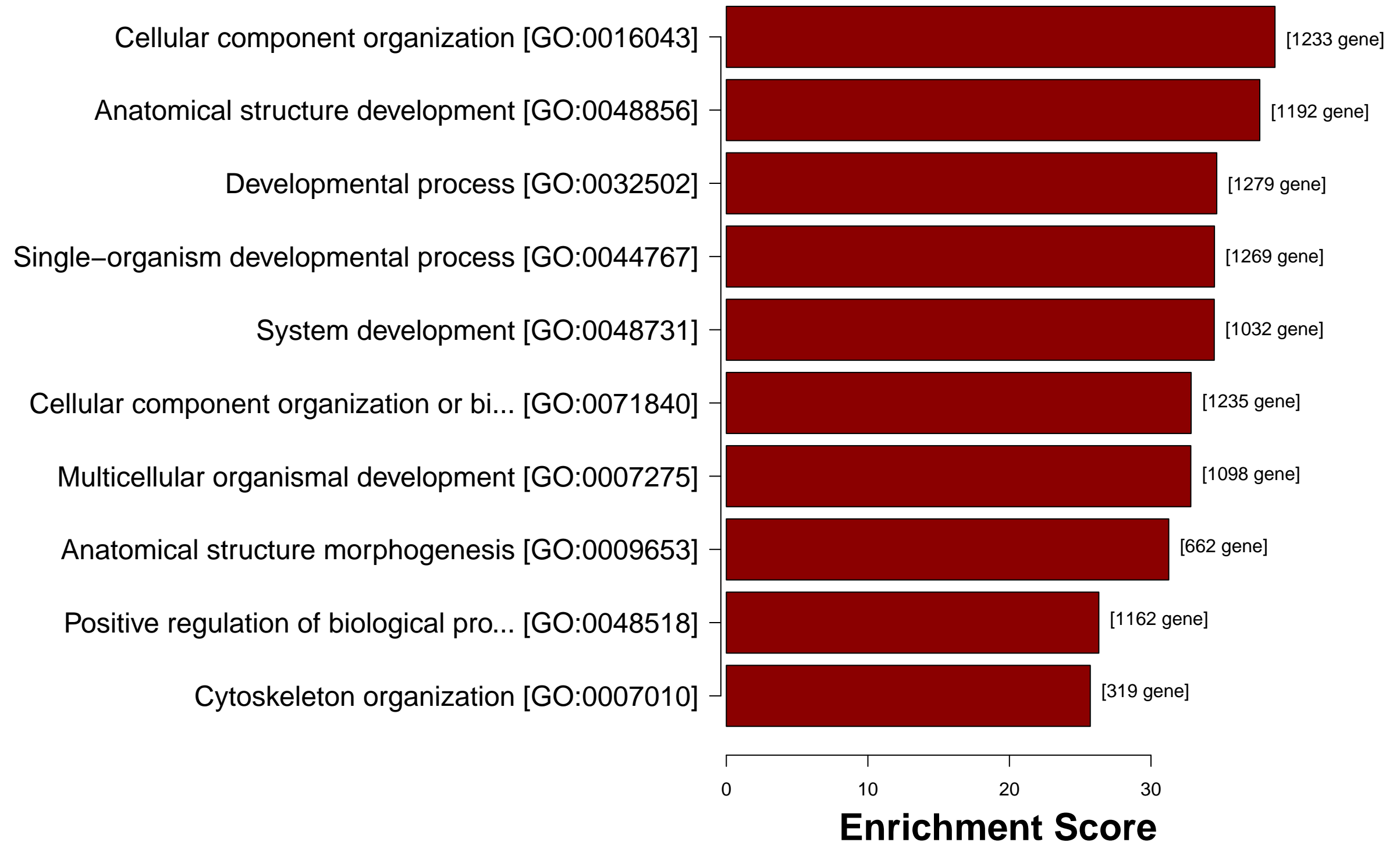

Supplement: Supplementary Materials — Figure S1: LPS-induced ARDS in rats. Sprague-Dawley rats were treated with LPS (20 mg/kg) via caudal vein 7 h before the lung tissues were collected, and the effect of LPS was assessed by histology in H&E-stained sections (bars = 100 μm; original magnification ×400). Figure S2: GO analysis of the biological function of genes. (A-B) Upregulated GO BP and CC terms for the DEGs were analyzed. Top 10 upregulated GO terms ranked by fold enrichment and enrichment score were shown. (C-D) Downregulated GO BP and CC terms for the DEGs were analyzed. Top 10 downregulated GO terms ranked by fold enrichment and enrichment score were shown. Figure S3: STRING analysis of interaction in the altered genes in rat ARDS. Nodes represent genes. Genes with more links are shown in a bigger size. Purple lines represent experimental evidence; yellow lines represent text-mining evidence; light lines represent database evidence. Table S1: the detailed characteristics of qRT-PCR for 11 candidate genes. Table S2: RNA-Seq reads and mapping rate of lung tissues from ARDS rats and normal rats. Table S3: upregulated KEGG pathway analysis. Table S4: downregulated KEGG pathway analysis. Supplemental Excel 1: a total of 5244 genes were considered to be significant, with the P value of <0.05 and FC > 1.5. Of those genes, 1413 genes were upregulated and 3831 downregulated. [file 4384797.f1.zip › 4384797.f1/Figure S2C2_BMRI_2212881.pdf]

# Sig GO terms of DE gene-CC

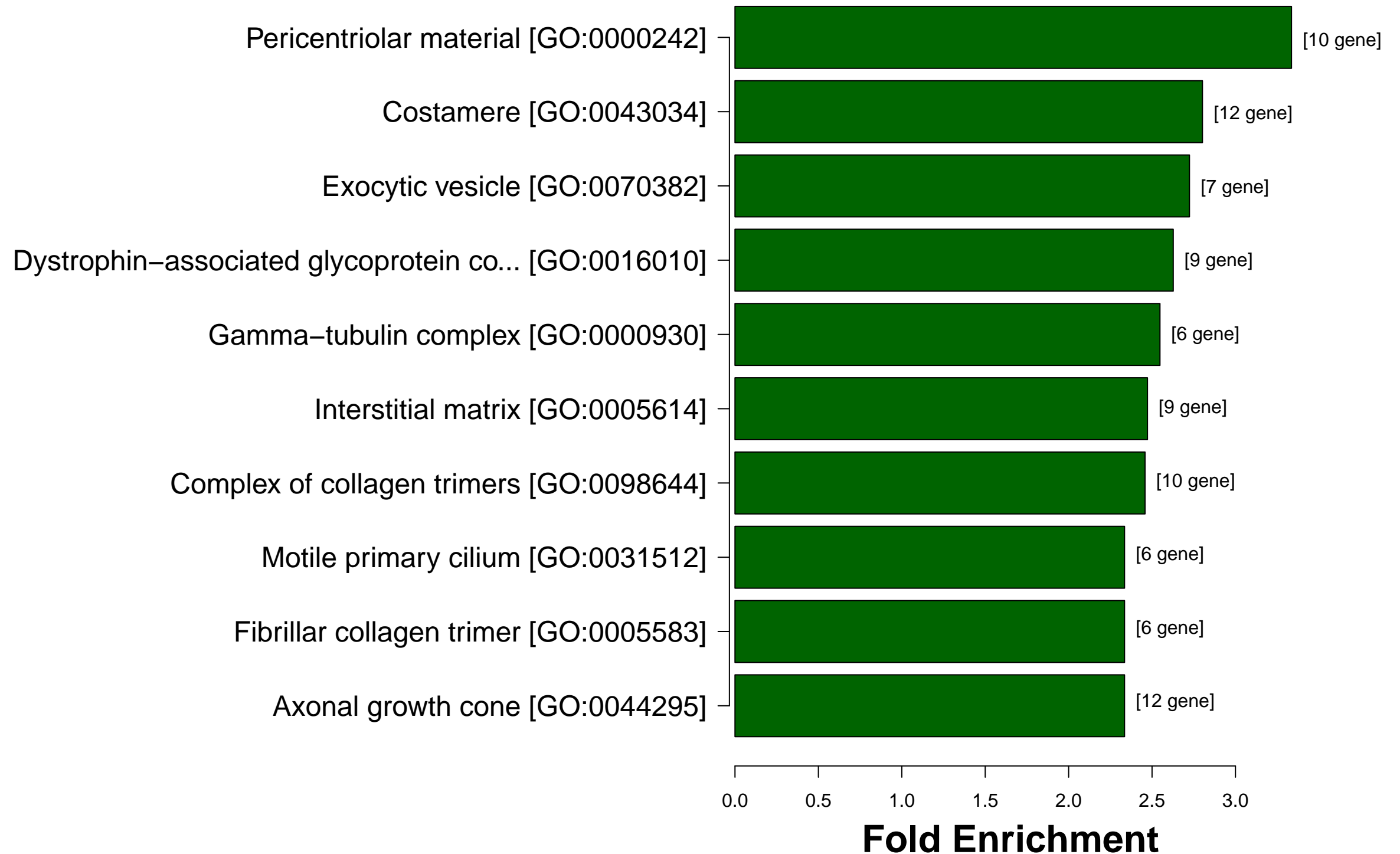

Supplement: Supplementary Materials — Figure S1: LPS-induced ARDS in rats. Sprague-Dawley rats were treated with LPS (20 mg/kg) via caudal vein 7 h before the lung tissues were collected, and the effect of LPS was assessed by histology in H&E-stained sections (bars = 100 μm; original magnification ×400). Figure S2: GO analysis of the biological function of genes. (A-B) Upregulated GO BP and CC terms for the DEGs were analyzed. Top 10 upregulated GO terms ranked by fold enrichment and enrichment score were shown. (C-D) Downregulated GO BP and CC terms for the DEGs were analyzed. Top 10 downregulated GO terms ranked by fold enrichment and enrichment score were shown. Figure S3: STRING analysis of interaction in the altered genes in rat ARDS. Nodes represent genes. Genes with more links are shown in a bigger size. Purple lines represent experimental evidence; yellow lines represent text-mining evidence; light lines represent database evidence. Table S1: the detailed characteristics of qRT-PCR for 11 candidate genes. Table S2: RNA-Seq reads and mapping rate of lung tissues from ARDS rats and normal rats. Table S3: upregulated KEGG pathway analysis. Table S4: downregulated KEGG pathway analysis. Supplemental Excel 1: a total of 5244 genes were considered to be significant, with the P value of <0.05 and FC > 1.5. Of those genes, 1413 genes were upregulated and 3831 downregulated. [file 4384797.f1.zip › 4384797.f1/Figure S2D1_BMRI_2212882.pdf]

# Sig GO terms of DE gene-CC

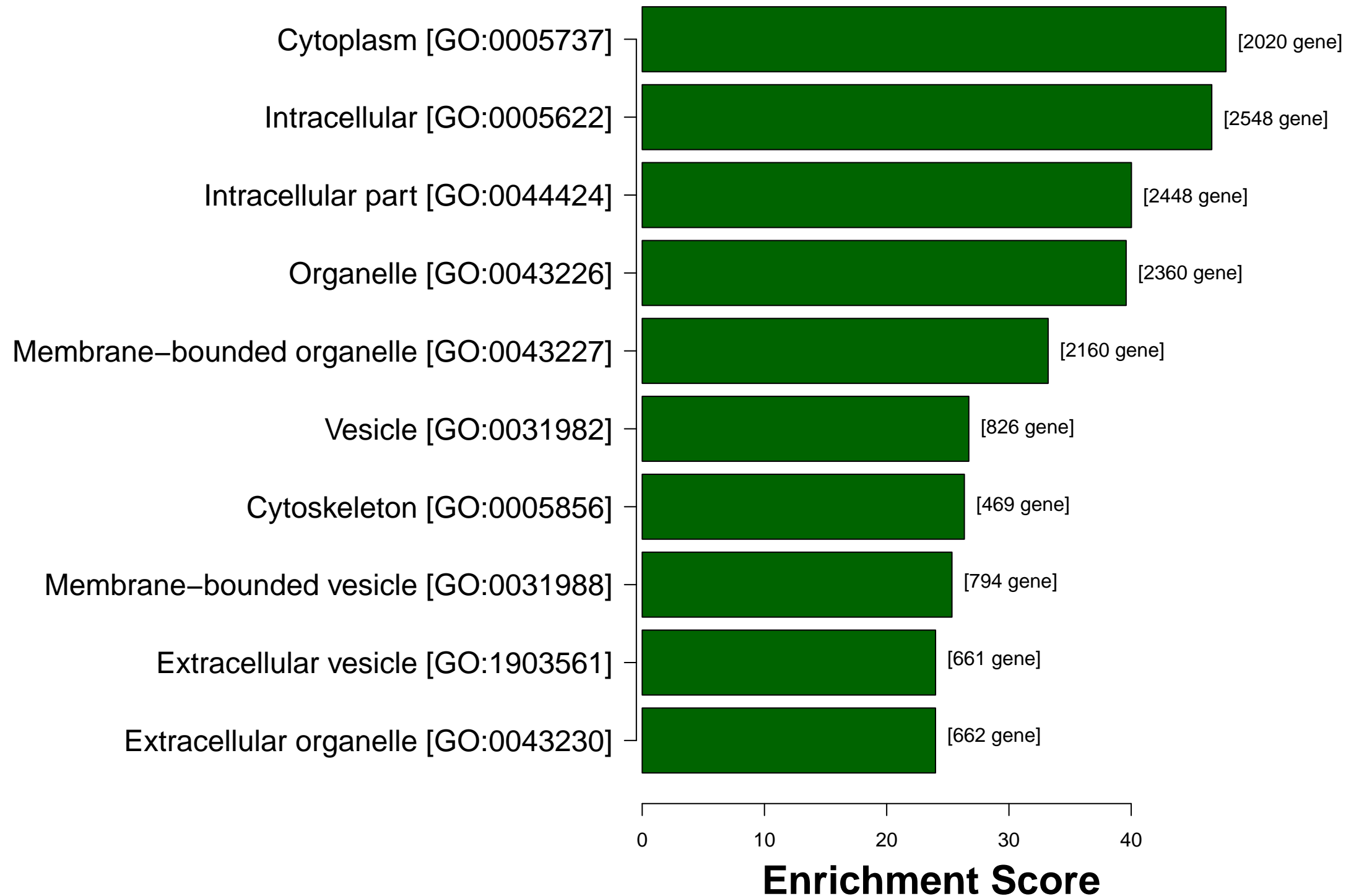

Supplement: Supplementary Materials — Figure S1: LPS-induced ARDS in rats. Sprague-Dawley rats were treated with LPS (20 mg/kg) via caudal vein 7 h before the lung tissues were collected, and the effect of LPS was assessed by histology in H&E-stained sections (bars = 100 μm; original magnification ×400). Figure S2: GO analysis of the biological function of genes. (A-B) Upregulated GO BP and CC terms for the DEGs were analyzed. Top 10 upregulated GO terms ranked by fold enrichment and enrichment score were shown. (C-D) Downregulated GO BP and CC terms for the DEGs were analyzed. Top 10 downregulated GO terms ranked by fold enrichment and enrichment score were shown. Figure S3: STRING analysis of interaction in the altered genes in rat ARDS. Nodes represent genes. Genes with more links are shown in a bigger size. Purple lines represent experimental evidence; yellow lines represent text-mining evidence; light lines represent database evidence. Table S1: the detailed characteristics of qRT-PCR for 11 candidate genes. Table S2: RNA-Seq reads and mapping rate of lung tissues from ARDS rats and normal rats. Table S3: upregulated KEGG pathway analysis. Table S4: downregulated KEGG pathway analysis. Supplemental Excel 1: a total of 5244 genes were considered to be significant, with the P value of <0.05 and FC > 1.5. Of those genes, 1413 genes were upregulated and 3831 downregulated. [file 4384797.f1.zip › 4384797.f1/Figure S2D2_BMRI_2212883.pdf]

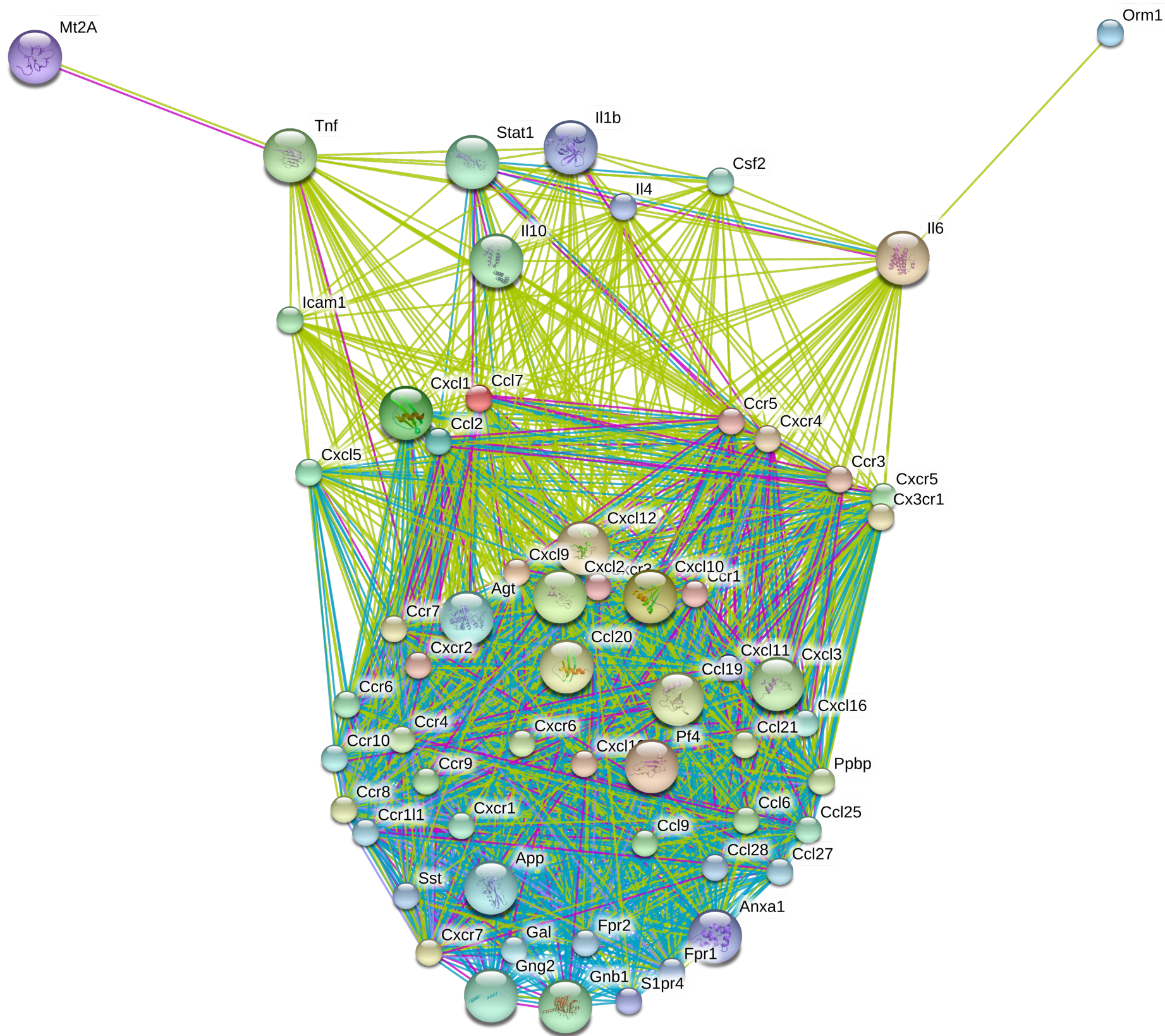

Supplement: Supplementary Materials — Figure S1: LPS-induced ARDS in rats. Sprague-Dawley rats were treated with LPS (20 mg/kg) via caudal vein 7 h before the lung tissues were collected, and the effect of LPS was assessed by histology in H&E-stained sections (bars = 100 μm; original magnification ×400). Figure S2: GO analysis of the biological function of genes. (A-B) Upregulated GO BP and CC terms for the DEGs were analyzed. Top 10 upregulated GO terms ranked by fold enrichment and enrichment score were shown. (C-D) Downregulated GO BP and CC terms for the DEGs were analyzed. Top 10 downregulated GO terms ranked by fold enrichment and enrichment score were shown. Figure S3: STRING analysis of interaction in the altered genes in rat ARDS. Nodes represent genes. Genes with more links are shown in a bigger size. Purple lines represent experimental evidence; yellow lines represent text-mining evidence; light lines represent database evidence. Table S1: the detailed characteristics of qRT-PCR for 11 candidate genes. Table S2: RNA-Seq reads and mapping rate of lung tissues from ARDS rats and normal rats. Table S3: upregulated KEGG pathway analysis. Table S4: downregulated KEGG pathway analysis. Supplemental Excel 1: a total of 5244 genes were considered to be significant, with the P value of <0.05 and FC > 1.5. Of those genes, 1413 genes were upregulated and 3831 downregulated. [file 4384797.f1.zip › 4384797.f1/Figure S3_BMRI_2212884.pdf]
